# Tumor vaccine composed of C-class CpG oligodeoxynucleotides and irradiated tumor cells induces long-term antitumor immunity

**DOI:** 10.1186/1471-2172-11-45

**Published:** 2010-09-13

**Authors:** Petra Cerkovnik, Barbara Jezersek Novakovic, Vida Stegel, Srdjan Novakovic

**Affiliations:** 1 Department of Molecular Diagnostics, Institute of Oncology Ljubljana, Zaloska 2, 1000 Ljubljana, Slovenia; 2 Department of Medical Oncology, Institute of Oncology Ljubljana, Zaloska 2, 1000 Ljubljana, Slovenia

## Abstract

**Background:**

An ideal tumor vaccine should activate both effector and memory immune response against tumor-specific antigens. Beside the CD8+ T cells that play a central role in the generation of a protective immune response and of long-term memory, dendritic cells (DCs) are important for the induction, coordination and regulation of the adaptive immune response. The DCs can conduct all of the elements of the immune orchestra and are therefore a fundamental target and tool for vaccination. The present study was aimed at assessing the ability of tumor vaccine composed of C-class CpG ODNs and irradiated melanoma tumor cells B16F1 followed by two additional injections of CpG ODNs to induce the generation of a functional long-term memory response in experimental tumor model in mice (i.p. B16F1).

**Results:**

It has been shown that the functional memory response in vaccinated mice persists for at least 60 days after the last vaccination. Repeated vaccination also improves the survival of experimental animals compared to single vaccination, whereas the proportion of animals totally protected from the development of aggressive i.p. B16F1 tumors after vaccination repeated three times varies between 88.9%-100.0%. Additionally, the long-term immune memory and tumor protection is maintained over a prolonged period of time of at least 8 months. Finally, it has been demonstrated that following the vaccination the tumor-specific memory cells predominantly reside in bone marrow and peritoneal tissue and are in a more active state than their splenic counterparts.

**Conclusions:**

In this study we demonstrated that tumor vaccine composed of C-class CpG ODNs and irradiated tumor cells followed by two additional injections of CpG ODNs induces a long-term immunity against aggressive B16F1 tumors.

## Background

Dendritic cells (DCs) play a crucial role in linking innate and adaptive immunity and consequently in the generation of a protective immune response against tumors [1]. The DCs as the most potent antigen presenting cells (APCs) are responsible for recognition and processing of tumor antigens, and they function as important initiators and modulators of the specific and lasting immune response against tumor antigens [1-3]. The ability of DCs to steer the immune response from the generation of effector and memory cells to the induction of peripheral tolerance is directed through their production of different cytokines such as TNF-α, IL-6, IL-12 and type I IFNs upon stimulation of specific receptors. In view of that the early signals given by the DCs can determine the scope and nature of the immune response [1,2]. The proper activation and maturation of the DCs is consequently crucial for the induction of an effective immune response against tumor cells. It has been demonstrated that "danger" signals are essential pre-requisites for the maturation and activation of DCs into powerful antigen presenting cells [1,3,4]. Synthetic CpG oligodeoxynucletides (CpG ODNs) contain unmethylated CpG motifs similar to those observed in bacterial DNA. They act as "danger" signals that trigger the maturation of DCs [5-7]. Through binding on Toll-like receptor 9 (TLR9) the CpG ODNs induce an elevated expression of MHC I and II molecules as well as of co-stimulatory molecules on DCs which in turn acquire an increased ability to present tumor antigens to T and B effector cells [8,9]. Besides, the CpG ODNs induce in the DCs and other APCs a secretion of different cytokines including TNF-α, IL-6, IL-12, IL-18 and IFNs (IFN-α, IFN-β) [1,10,11]. In this way, the CpG ODNs indirectly activate natural killer (NK) cells and cytotoxic T lymphocytes (CTL) in addition to antigen-specific, antibody-producing B cells.

Among different classes of CpG ODNs with immuno-stimulatory activities, the B-class CpG ODNs (which are strong B cell stimulators but poor inducers of IFN-α in DCs) are the ones used in nearly all of the vaccine studies performed to date and are more advanced in clinical application in oncology being tested in phase II and phase III clinical trials as cancer vaccine adjuvant and in combined therapies [6,10-16]. However, C-class CpG ODNs (that stimulate B cells and induce intensive type I IFN production by DCs) have also shown the vaccine adjuvant activity [17,18]. For example, in study performed by Wille-Reece, the nonhuman primates vaccinated with HIV Gag protein/Montanide and C-class CpG ODNs or the TLR7/8 agonist (3M-012) had higher frequencies of Th1 response after primary immunization compared to other vaccine groups immunized only with HIV Gag protein/Montanide or with HIV Gag protein/Montanide and TLR8 agonist 3M-002 [17]. In our previous study, we demonstrated a significant preventive anti-tumor immunity achieved through the vaccination with irradiated melanoma tumor cells B16F1 and C-class CpG ODNs followed by two additional injections of CpG ODNs. The proportion of protected mice ranged from 75% to 100%. Additionally, in more than 80% of survivors, a long-lasting immunity has been triggered [19]. Class C CpG ODNs were also shown to suppress the growth of s.c. B16F1 tumors when applied as a single agent and to remarkably enhance the anti-tumor effect of tumor irradiation in combined therapy [20].

The determination of the manner by which the long-lived cellular immune response is generated following vaccination and of the mechanism by which memory cells are maintained over time is important in the development of a safe and effective tumor vaccine. In this article, we sought to investigate whether the tumor vaccine composed of C-class CpG ODNs and irradiated melanoma B16F1 tumor cells followed by two additional injections of CpG ODNs could induce a functional long-term memory response. Additionally, we evaluated the duration of the long-lasting protection as well as the distribution and homing of the long-lived memory cells after immunization with different vaccination settings. For this purpose we chose a weakly immunogenic B16F1 tumor model.

## Results

### The achieved antitumor protection in repeatedly vaccinated mice lasts at least 60 days

**Figure 1:**
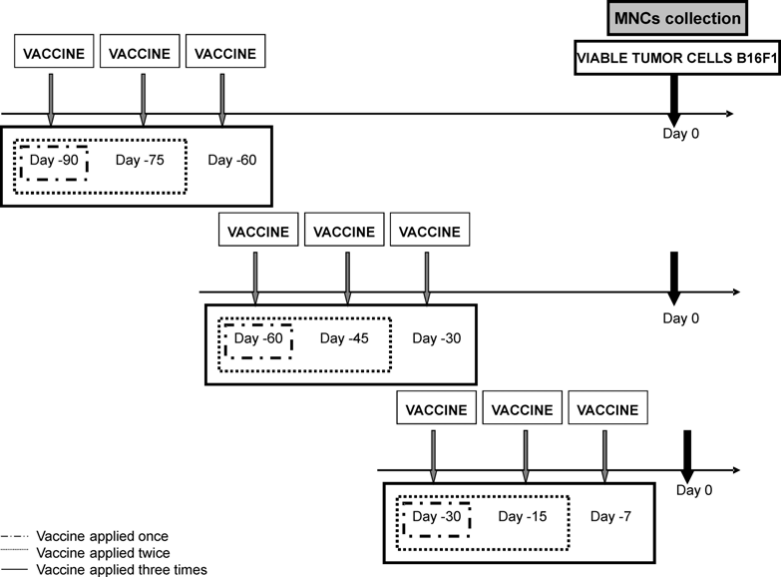
**Time schedule of i.p. applications of the tumor vaccine and viable tumor cells**. The day of MNCs collection is indicated.

In this part, we investigated the longevity of the induced antitumor protection. For that purpose, the experimental mice had been once, twice or three times pre-vaccinated 90, 60 or 30 days before they were challenged with viable tumor cells (day 0) (see Figure 1).

**Figure 2:**
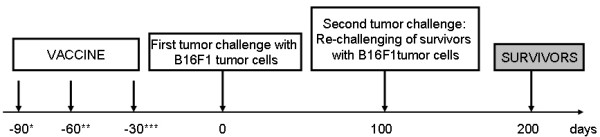
**Time schedule of re-challenge with 5 × 10**^**5 **^**viable B16F1 tumor cells of pre-vaccinated mice surviving the first tumor challenge**. * vaccination started 90 days before the first injection of viable tumor cells; the vaccine was applied once (1×), twice (2×) or three times (3×) in 15 days interval ** vaccination started 60 days before the first injection of viable tumor cells; the vaccine was applied once (1×), twice (2×) or three times (3×) in 15 days interval *** vaccination started 30 days before the first injection of viable tumor cells; the vaccine was applied once (1×), twice (2×) in 15 days interval or three times (3×) in 15 days interval, except for the last vaccination, which was performed 7 days after second vaccination.

The average survival was statistically significantly prolonged after the application of the tumor vaccine and two additional injections of CpG ODNs in all experimental groups compared to the mock treated control group (p < 0.001; Figure 2; Table 1). In a fraction of mice that had been vaccinated twice and three times, the antitumor protection was upgraded in comparison to mice immunized only once. Namely, the proportion of protected mice ranged from 28.6% to 38.1% in case of a single vaccination and it increased to 68.2% - 75.0% in groups vaccinated twice and to 88.9%-100.0% in groups vaccinated three times (Table 1). The day of the first tumor vaccine application did not influence the survival of animals significantly. Convincing antitumor preventive effect was accomplished with the triple (applied three times) vaccination irrespective of the day of first vaccine application (p = 0.377).

**Table 1:** The antitumor preventive effect of the tumor vaccine composed of irradiated tumor cells and CpG ODNs.

Group	**Number of mice**^**a**^	**Number of survivors **^**b**^	Proportion of survivors (%)	Average survival AM ± SE (days)	**p-value**^**c**^	**p-value**^**d**^
Control	22	0	0.0	16.09 ± 1.14		
1 × 90d*	21	8	38.1	60.38 ± 8.21	<0.0001	
2 × 90d*	20	14	75.0	85.30 ± 5.97	<0.0001	0.012
3 × 90d*	21	19	90.5	94.38 ± 3.93	<0.0001	0.003
1 × 60d**	21	6	28.6	51.00 ± 7.86	<0.0001	
2 × 60d**	22	16	72.7	82.82 ± 6.69	<0.0001	0.002
3 × 60d**	18	16	88.9	89.10 ± 5.12	<0.0001	<0.001
1 × 30d***	22	7	31.8	62.14 ± 7.60	<0.0001	
2 × 30d***	22	15	68.2	81.00 ± 6.66	<0.0001	0.022
3 × 30d***	20	20	100.0	100.00 ± 0.00	<0.0001	0.003

The vaccine was composed of 1 × 10^6 ^irradiated B16F1 tumor cells and 30 μg of CpG ODNs; each vaccine administration was followed by two additional injections of CpG ODNs (2 and 4 days after vaccine administration).

^a ^number of animals included in the experimental group

^b ^surviving animals - are animals which survived at least 100 days after tumor challenge

^c ^p-value: group compared to control group

^d ^p-value: group compared to group vaccinated once starting the same day prior to tumor challenge

* vaccination started 90 days before the injection of viable tumor cells; the vaccine was applied once (1×), twice (2×) or three times (3×) in 15 days interval

** vaccination started 60 days before the injection of viable tumor cells; the vaccine was applied once (1×), twice (2×) or three times (3×) in 15 days interval

*** vaccination started 30 days before the injection of viable tumor cells; the vaccine was applied once (1×), twice (2×) in 15 days interval or three times (3×) in 15 days interval, except for the last vaccination, which was performed 7 days after second vaccination

The experiment was repeated three times.

The vaccine (followed by two additional injections of CpG ODNs) applied two or three times also significantly prolonged the survival of mice in comparison to a single vaccination (p = 0.023; p < 0.001 respectively; Table 1). Again the most persuasive effect was achieved with the vaccine applied three times irrespective of the day of first application. There was no difference in survival between the triple vaccination starting 90 days before tumor challenge and the triple vaccination starting 30 days before tumor challenge (p = 0.413).

Taken together, our results show that the vaccine must be applied at least two times to achieve a functional memory response and subsequently the tumor rejection.

### The long-lasting protective antitumor immunity is generated in survivors

**Figure 3:**
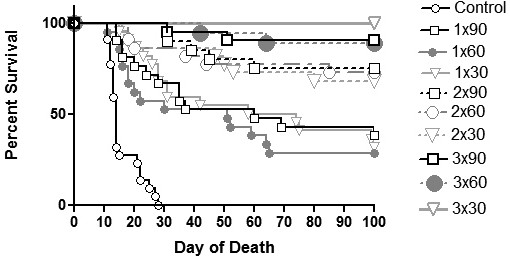
**Survival of vaccinated mice challenged i.p. with 5 × 10**^**5 **^**viable B16F1 tumor cells**. Mice were immunized with tumor vaccine composed of 1 × 10^6 ^B16F1 irradiated tumor cells and 30 μg of CpG ODNs, followed by two additional injections of 30 μg of CpG ODNs 2 and 4 days after tumor vaccine application. The tumor vaccine was first applied on day 90, day 60 or day 30 prior to viable tumor cell inoculation (day 0). The tumor vaccine was administrated once (1×), twice (2×) and three times (3×) in 15 days period. Control: mock treated; 1 × 90: vaccine + two additional injections of CpG ODNs applied once 90 days before tumor inoculation; 2 × 90: vaccine + two additional injections of CpG ODNs applied twice 90 and 75 days before tumor inoculation; 3 × 90: vaccine + two additional injections of CpG ODNs applied three times 90, 75 and 60 days before tumor inoculation; 1 × 60: vaccine + two additional injections of CpG ODNs applied once 60 days before tumor inoculation; 2 × 60: vaccine + two additional injections of CpG ODNs applied twice 60 and 45 days before tumor inoculation; 3 × 60: vaccine + two additional injections of CpG ODNs applied three times 60, 45 and 30 days before tumor inoculation; 1 × 30: vaccine + two additional injections of CpG ODNs applied once 30 days before tumor inoculation; 2 × 30: vaccine + two additional injections of CpG ODNs applied twice 30 and 15 days before tumor inoculation; 3 × 30: vaccine + two additional injections of CpG ODNs applied three times 30, 15 and 7 days before tumor inoculation. The experiment was repeated three times.

**Figure 4:**
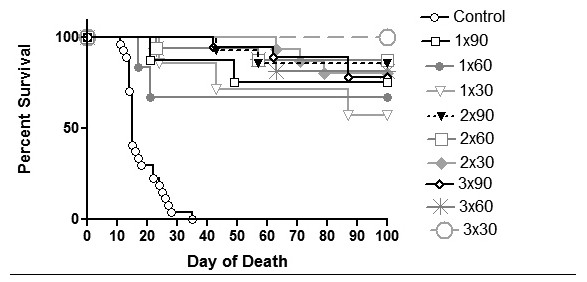
**Survival curves of mice re-challenged i.p. with 5 × 10**^**5 **^**viable B16F1 tumor cells**. Pre-vaccinated mice surviving the first tumor challenge were without any additional treatment re-challenged 100 days after the first tumor challenge. Control: mock treated; 1 × 90: vaccine + two additional injections of CpG ODNs applied once 90 days before tumor inoculation; 2 × 90: vaccine + two additional injections of CpG ODNs applied twice 90 and 75 days before tumor inoculation; 3 × 90: vaccine + two additional injections of CpG ODNs applied three times 90, 75 and 60 days before tumor inoculation; 1 × 60: vaccine + two additional injections of CpG ODNs applied once 60 days before tumor inoculation; 2 × 60: vaccine + two additional injections of CpG ODNs applied twice 60 and 45 days before tumor inoculation; 3 × 60: vaccine + two additional injections of CpG ODNs applied three times 60, 45 and 30 days before tumor inoculation; 1 × 30: vaccine + two additional injections of CpG ODNs applied once 30 days before tumor inoculation; 2 × 30: vaccine + two additional injections of CpG ODNs applied twice 30 and 15 days before tumor inoculation; 3 × 30: vaccine + two additional injections of CpG ODNs applied three times 30, 15 and 7 days before tumor inoculation. The experiment was repeated three times.

In order to answer the question for how long the pre-vaccinated mice are protected against the tumorigenic effect of viable tumor cells, mice that had been preventively vaccinated and had already survived one tumor challenge were re-challenged with viable tumor cells 100 days after the first tumor challenge without any additional treatment (see Figure 3). It is obvious from Table 2 and Figure 4 that in more than 60.0% of mice having survived the first tumor challenge the long-lasting immunity was induced. The proportion of survivors after the second tumor challenge was higher in mice vaccinated two or three times (at least 75.0%) yet not significantly when compared to a single vaccination (p = 0.452). Hence, the proportions of survivors were evenly distributed among the experimental groups. These results suggest that memory cells specific for the tumor antigens persist in vaccinated animals for at least 8 months following the immunization with tumor vaccine and two additional injections of CpG ODNs.

**Table 2:** Long-term protection of mice surviving the i.p. challenge with viable tumor cells.

Group		**Number of mice**^**a**^		**Proportion of survivors (%)**^**b**^	Average survival AM ± SE (days)	**p-value**^**c**^
Control		27		0.0	18.00 ± 1.14	
1 × 90d*		8		75.0	83.75 ± 10.96	<0.0001
2 × 90d*		14		85.7	92.86 ± 4.91	<0.0001
3 × 90d*		19		73.7	90.89 ± 4.25	<0.0001
1 × 60d**		6		66.7	73.00 ± 17.08	<0.0001
2 × 60d**		16		87.5	92.50 ± 5.35	<0.0001
3 × 60d**		16		81.3	90.19 ± 5,63	<0.0001
1 × 30d***		7		57.1	79.14 ± 12.10	<0.0001
2 × 30d***		15		80.0	94.20 ± 12.38	<0.0001
3 × 30d***		20		100.0	100.00 ± 0.00	<0.0001

Mice surviving the i.p. challenge with 5 × 10^5 ^B16F1 viable tumor cells were re-challenged with the same number of viable tumor cells without additional treatment.

^a ^number of animals which survived 100 days after first tumor challenge

^b ^surviving animals - are animals which survived at least 100 days after tumor challenge

^c ^p-value: group compared to control group

* vaccination started 90 days before the injection of viable tumor cells; the vaccine was applied once (1×), twice (2×) or three times (3×) in 15 days interval

** vaccination started 60 days before the injection of viable tumor cells; the vaccine was applied once (1×), twice (2×) or three times (3×) in 15 days interval

*** vaccination started 30 days before the injection of viable tumor cells; the vaccine was applied once (1×), twice (2×) in 15 days interval or three times (3×) in 15 days interval, except for the last vaccination, which was performed 7 days after second vaccination

The experiment was repeated three times.

### Long-lived memory T cells home predominantly to bone marrow and to peritoneal tissue

In this part, we investigated the activation and specificity of the memory response along with the distribution of memory cells after vaccination. For this purpose, the MNCs were isolated from bone marrow, spleen and peritoneal lavage. They were grown for additional 5 days with or without tumor antigens (irradiated B16F1 tumor cells) to have ultimately their cytotoxicity against B16F1 tumor cells assessed by cytotoxicity assay.

**Figure 5:**
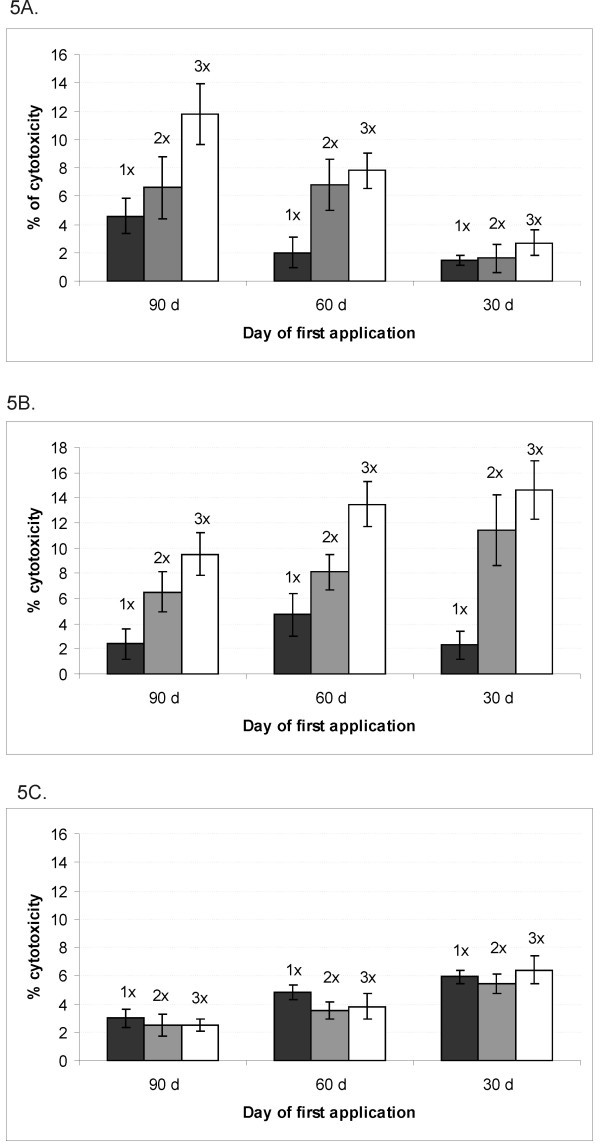
**The cytotoxicitiy of MNCs (AM ± SD) isolated from bone marrow (A), peritoneal lavage (B) and spleen (C)**. Bone marrow, peritoneal lavages and spleens from pre-vaccinated mice were collected on day 0 and subsequently MNCs were isolated. Half of the MNCs from each experimental group were re-exposed to the antigen - irradiated B16F1 tumor cells and another half was grown without any additional stimulation for 5 days. The cytotoxicity of MNCs was determined as described in Materials and methods and was normalized against the control (mock) treated group. 1×: vaccine + two additional injections of CpG ODNs applied once 90, 60 or 30 days before tumor inoculation; 2×: vaccine + two additional injections of CpG ODNs applied twice 90 and 75 days before tumor inoculation, 60 and 45 days before tumor inoculation or 30 and 15 days before tumor inoculation; 3×: vaccine + two additional injections of CpG ODNs applied three times 90, 75 and 60 days before tumor inoculation, 60, 45 and 30 days before tumor inoculation or 30, 15 and 7 days before tumor inoculation.

The data presented in Figure 5A indicate that the cytotoxicity of MNCs isolated from bone marrow was increased in mice vaccinated 90 and 60 days before tumor challenge compared to mice vaccinated 30 days before tumor challenge. The cytotoxicity of bone marrow MNCs was significantly upgraded after two or three vaccinations starting 90 or 60 days before tumor challenge in comparison to mice vaccinated two or three times starting 30 days before tumor challenge (Table 3). Additionally, in mice receiving the vaccine three times starting 90 or 60 days before tumor challenge, a statistically significant augmentation of cytotoxicity was determined compared to a single vaccination starting at the same time (p = 0.007; p = 0.004 respectively). There was also a significant difference between the cytotoxicity of MNCs from mice treated with the tumor vaccine twice and cytotoxicity of MNCs from mice vaccinated three times starting on day 90 (p = 0.043), whereas this difference was not observed in mice starting the vaccination on day 60 or 30 prior to tumor challenge (p = 0.474; p = 0.071 respectively).

**Table 3:** The significantly increased cytotoxicity of bone marrow MNCs obtained from mice pre-vaccinated 90 and 60 days prior to MNCs collection.

Group	**p-value**^**a**^	**p-value**^**b**^	**p-value**^**c**^
1 × 90d*	0.014	0.031	0.011
2 × 90d*	0.016	0.023	0.002
3 × 90d*	0.001	0.002	<0.001
1 × 60d**	0.478	0.664	0.245
2 × 60d**	0.007	0.012	<0.001
3 × 60d**	0.001	0.003	<0.001

^a ^p-value: group compared to group vaccinated once 30 days before tumor challenge

^b ^p-value: group compared to group vaccinated twice: 30 and 15 days before tumor challenge

^c ^p-value: group compared to group vaccinated three times: 30, 15 and 7 days before tumor challenge

* vaccination started 90 days before the injection of viable tumor cells; the vaccine was applied once (1×), twice (2×) or three times (3×) in 15 days interval

** vaccination started 60 days before the injection of viable tumor cells; the vaccine was applied once (1×), twice (2×) or three times (3×) in 15 days interval

In Figure 5B, the cytotoxicity of MNCs isolated from peritoneal cavity is presented. The cytotoxicity of these cells was enhanced by increasing the number of vaccinations irrespectively of the day of first vaccine application. The statistically significant enhancement of cytotoxicity was determined when tumor vaccine was applied two and three times starting 90, 60 or 30 days prior to tumor challenge in comparison to the cytotoxicity of MNCs obtained from mice vaccinated just once (Table 4).

**Table 4:** The cytotoxicity of MNCs from peritoneal tissue was significantly enhanced after repeated vaccinations.

Group	2 × 90d*	3 × 90d*	2 × 60d**	3 × 60d**	2 × 30d***	3 × 30d***
**p-value **^**a**^	0.024	0.004	0.056	0.004	0.006	<0.001

^a ^p-value: group compared to group vaccinated once 90, 60 or 30 days before tumor challenge

* vaccination started 90 days before the injection of viable tumor cells; the vaccine was applied once (1×), twice (2×) or three times (3×) in 15 days interval

** vaccination started 60 days before the injection of viable tumor cells; the vaccine was applied once (1×), twice (2×) or three times (3×) in 15 days interval

*** vaccination started 30 days before the injection of viable tumor cells; the vaccine was applied once (1×), twice (2×) or three times (3×) in 15 days interval, except for the last - third vaccination, which was performed 7 days after second vaccination

The Figure 5C shows the cytotoxicity of splenic MNCs. The cytotoxicity was decreased with the longer time interval between the first day of vaccination and MNCs collection, since the cytotoxicity in mice vaccinated 90 days before MNCs collection was significantly reduced compared to mice vaccinated 30 days before MNCs collection (p = 0.003). The cytotoxicity of splenic MNCs was unaffected by the number of vaccinations. Nevertheless, the cytotoxicity of splenic MNCs was inferior to the cytotoxicity of MNCs isolated from bone marrow or peritoneal lavage (p = 0.002).

The cytotoxicity assay confirms that the functional memory is generated after two and three vaccinations. Memory cells predominantly home to bone marrow and to peritoneal tissue.

## Discussion

In our previous study we demonstrated that significant antitumor immunity was generated through the vaccination of mice with CpG ODNs in combination with irradiated tumor cells (tumor vaccine) followed by two additional injections of CpG ODNs. We confirmed also that for the improvement of the *in vivo *effect additional injections of CpG ODNs in form of maintenance monotherapy are essential. A long-lasting presence of CpG ODNs is therefore required for assuring the maturation of APCs during a longer period of time and the required signal for maturation of an adequate number of DCs can be provided only by prolongation of treatment with CpG ODNs [19].

The present study was aimed to investigate whether the tumor vaccine composed of C-class CpG ODNs and irradiated melanoma B16F1 tumor cells followed by two additional injections of CpG ODNs could induce a functional long-term memory response. Further we planned to evaluate the duration of the long-lasting protection as well as the distribution and homing of long-lived memory cells after immunization with different vaccination settings. With the initial experiments we confirmed that a functional memory response is induced upon vaccination with tumor vaccine composed of irradiated B16F1 tumor cells and C-class CpG ODNs followed by two additional injections of CpG ODNs. A substantial antitumor immunity was achieved in all experimental mice immunized with the tumor vaccine compared to control (mock treated) mice (p < 0.001). The induced long-lasting preventive effect was additionally improved when the vaccination with CpG ODNs in combination with irradiated tumor cells was repeated two or three times. The proportion of animals totally protected from the development of aggressive i.p. B16F1 tumors after vaccination repeated three times ranged from 88.9% to 100.0%. These results suggested that the repeated vaccination was more effective in generating a durable antitumor response than a single vaccination. Similarly, in the study of Wu and Fleischmann, it was reported that after repeated vaccination of mice with a tumor vaccine (composed of irradiated B16 melanoma cells exposed *in vitro *to long-term IFN-alpha treatment) a protective durable immunity against a second tumor challenge was significantly increased [21,22]. Our results are also in agreement with the statements of other authors that short-term or weak antigen stimulation can trigger the initial proliferation of effector T cells but is insufficient to trigger the long-lived memory T cells [23-25]. On the other hand, the higher number of vaccinations resulted in a repeatedly longer lasting presence of CpG ODNs, which were in turn able to activate a sufficient number of APCs capable of inducing the memory cell development.

The duration of immune response was explored from different views. Firstly - by changing the interval between vaccination and challenge with viable tumor cells. Secondly - by changing the number of vaccine applications and thirdly - by assessing the effectiveness of immune response in surviving mice without additional vaccination. Our results showed no difference in survival of mice being vaccinated three times but starting at different days: 90, 60 or 30 days before tumor challenge (p = 0.413). Yet, significantly longer survival was observed among mice vaccinated two or three times and those vaccinated just once. These outcomes suggest that a strong antitumor immunity in vaccinated mice persists for at least 60 days after the last vaccination. Our results are also in accordance with results reported by Mahnke et al., who showed that 66.0% of vaccinated nude mice did not develop tumors after high-dose i.v. tumor challenge more than 2 months after adoptive immunotherapy [26]. The cells involved in long-term immune protection were memory cells by definition as tumor challenge occurred more than 2 months after the initial effector phase had subsided. It was confirmed that the maintenance of functional long-term CD8+ T-cell memory is independent of antigen persistence, although the continuous presence of antigen results in the boosting of antigen-specific T-cell numbers [26]. In our study, the repeated vaccinations might have favored the generation and maintenance of tumor-specific CD8+ T-cell memory pools.

The next question in the study was about the duration of the antitumor prevention assured by the vaccination. Addressing this question, the experimental mice that had survived the first tumor challenge were re-challenged with the same type of viable tumor cells (100 days after first tumor challenge) without additional treatment. We observed that in more than 60.0% of survivors a long-lasting immunity was induced. The number of survivors developing the long-lasting immunity did not depend upon the number of repeated vaccinations. These results indicate that the activation of a critical number of APCs had been accomplished when mice were treated with the vaccine composed of irradiated tumor cells and C-class CpG ODNs and two additional applications of CpG ODNs. The first functional response and the proliferative expansion of T lymphocytes after the vaccination were accompanied by the differentiation of specific memory T lymphocytes functioning as long-term memory T cells and enabling the long-lasting immunity [19]. Our results suggest that the long-term immune memory and tumor protection could be maintained over a prolonged period of time - at least 8 months. The comparable duration of long term memory and tumor protection after adoptive transfer of memory peritoneal exudate cells from donors to recipients was earlier reported by Mahnke et al. [26].

In the last part of our experiments, we investigated the homing place of memory T cell pool following vaccination. Using the cytotoxicity assay, we confirmed that MNCs isolated from bone marrow and peritoneal lavage are more cytotoxic against B16F1 tumor cells than their counterparts in spleen. This is in agreement with the reports of other investigators, who described a higher frequency and higher cytotoxicity of antigen-specific memory T cells in the bone marrow compared to spleen, lymph nodes or peripheral blood [26-28]. Mahnke et al. showed that the bone marrow microenvironment has special features that are of importance for the maintenance of tumor dormancy and immunological T-cell memory, and that a low level of persisting antigen favors the maintenance of antigen-specific memory T cells over irrelevant memory T cells [26]. On the other hand, Parretta et al. demonstrated that the bone marrow acts as a suitable microenvironment for the antigen-independent proliferation of memory CD8+ cells (primarily cytokine driven) and plays a relevant role in the maintenance of T cell memory [28]. The presence of antitumor CTLs in the bone marrow of untreated breast cancer patients which has been associated with the local control of micrometastasis growth might also be due to the preferential maintenance of memory CD8+ cells in the bone marrow [28]. In the light of above findings, our results revealed that after a prolonged period following the vaccination (2-3 months) memory cells from the bone marrow and peritoneal tissue were in a more active state than those from the spleen. This could firstly be explained by the observation that in the bone marrow memory CD8+ cells might receive survival/proliferation signals sustaining the long-term maintenance of these cells. Indeed, the stromal cells and cells of the hematopoietic lineage in the bone marrow produce both IL-7 and IL-15 that stimulate the CD8+ cell survival and proliferation [28,29]. Secondly, it was suggested that the optimal preservation of T-cell memory requires the presence of the DCs. Cavanagh et al. showed that a small number of differentiated DCs traffics constitutively from peripheral tissue to blood and that these circulating DCs have a considerable bone marrow tropism. Once in the bone marrow, the DCs induce a rapid proliferation of antigen-specific memory T cells [30]. In this view, the repeated vaccination results in a higher number of mature, tumor antigen-bearing DCs and therefore could act in "boosting" of memory responses against tumor cells.

It is recognized that memory T cells are heterogeneous in terms of both homing capacity and effector function [31,32]. This heterogeneity is reflected in the current definition of central memory and effector memory T cells [31,32]. Effector memory T cells home to peripheral tissues, they rapidly produce effector cytokines such as IFN-γ upon antigenic stimulation, but have limited proliferative capacity. Our results indicate that following immunization with tumor vaccine the migration of memory T cells to the bone marrow bears obvious parallels with homing of memory T-cell into peritoneal tissue. Recent reports have shown high levels of constitutive memory T-cell lodging into extra-lymphoid organs in the absence of overt inflammation, and it has been demonstrated that epithelial cells produce homeostatic chemokines able to attract T lymphocytes at the body surfaces [29]. Finally, our *in vitro *results confirmed the results from *in vivo *experiments. The tumor vaccine applied two or three times significantly increased the cytotoxicity of MNCs isolated from bone marrow and peritoneal lavage compared to single vaccination. It is likely that the repeated injections of tumor antigens in combination with CpG ODNs result in the boosting of tumor-specific T-cell numbers and consequently in memory cell pool enhancement. Opposingly, the cytotoxicity of splenic MNCs was not significantly influenced by the number of vaccinations indicating again that the bone marrow is the superior compartment for harboring of tumor-specific memory cells. This correlates with previous reports of memory T-cell enrichment in the bone marrow of experimental animals and cancer patients [33] and also with a higher extent of retention of circulating DCs in the bone marrow compared to spleen [30].

## Conclusions

In conclusion, we demonstrated that tumor vaccine composed of C-class CpG ODNs and irradiated tumor cells followed by two additional injections of CpG ODNs induces a long-term immunity against aggressive B16F1 tumors. Repeated vaccination improves the survival of experimental animals compared to a single vaccination. A functional response is induced for at least 60 days and the long-term immune memory and tumor protection can be maintained over a prolonged period of time (at least 8 months). Following vaccination, tumor-specific memory cells predominantly reside in bone marrow and peritoneal tissue being in a more active state than their splenic counterparts.

## Methods

### Cell lines

Murine B16F1 melanoma cells (American Type Culture Collection, ATCC, Rockville, MD) were grown in Eagle's minimal essential medium (EMEM) supplemented with 10% FCS (Sigma St. Louis, MO), penicillin (100 units/ml, Pfizer, New York, NY), streptomycin (100 μg/ml, Pfizer) and gentamycin (11 μg/ml, Invenex, Charing Falls, OH).

### Animal tumor model

The experiments were performed on 8 - 10 weeks old syngeneic female C57Bl/6 mice (Institute of Pathology, University of Ljubljana, SLO). Experimental animals were kept in standard animal colony at a natural day/night cycle. At least 6 healthy animals without signs of fungal or other infections, and with normal body weight, were included in each experimental group. Intraperitoneal (i.p.) B16F1 tumor model was employed. Tumors were induced by i.p. inoculation of 5 × 10^5 ^viable B16F1 tumor cells in 0.2 ml EMEM supplemented with 2% FCS. The day of tumor cells inoculation was considered as day 0. The animals were monitored for the day of death and the proportion of survivors was noted. The average survival (AM) ± standard deviation (SD) ± standard error (SE) was calculated for the animals that ultimately developed tumors and consequently died of them. The experiment was performed (repeated) three times.

The experiments were approved by the Ministry of Agriculture, Forestry and Food of the Republic of Slovenia (permission No. 34401-35/2008/12).

### Vaccine preparation

Tumor vaccine was composed of 1 × 10^6 ^B16F1 irradiated tumor cells and 30 μg of CpG ODN 2395 class C (Coley Pharmaceutical, Ontario, CA) per mouse.

Tumor cell preparation: B16F1 tumor cells were trypsinized (0.25% trypsin, Sigma) and washed three times in the 10% serum-containing medium. The tumor pellets were than resuspended in the 2% serum containing EMEM (in concentration of 1 × 10^6 ^cells/cm^2^) and irradiated sublethally with 60 Gy on Darpac 2000x X-ray unit (Gulmay Medical Ltd., Shepperton, UK). Tumor cells, which were neither clonogenic *in vitro*, nor tumorigenic *in vivo *were taken as sublethally irradiated.

### Vaccine and CpG ODN administration

The experimental mice were treated with the tumor vaccine i.p. followed by two additional injections of 30 μg of CpG ODNs (i.p.) 2 and 4 days after vaccine administration. The treatment schedule was as follows: the vaccine followed by two additional CpG ODNs applications was applied once, twice or three times starting on days 90, 60 or 30 prior to the injection of viable tumor cells (day 0). When the vaccine was administered two or three times the interval between the administrations was 15 days, except in the case of last administration in the group of animals treated three times beginning on day 30 - this administration was just 7 days after the second administration. (Figure 1).

### Evaluation of long-lasting immunity

Mice that have been preventively treated with the vaccine in combination with two additional doses of CpG ODN were challenged i.p. with 5 × 10^5 ^viable B16F1 tumor cells. After 100 days, the survivors were re-challenged with 5 × 10^5 ^viable tumor cells without additional pre-vaccination (Figure 3).

### Isolation of mouse MNCs from spleen, bone marrow and peritoneal lavage

The spleens, femurs and peritoneal lavages were collected on day 0 (Figure 1). The spleens were cut in small pieces and mechanically disrupted. The bone marrow was rinsed with EMEM from the femurs. The peritoneal cavity was rinsed two times with PBS and peritoneal lavage was than centrifuged at 1500 rpm for 5 min and cell pellets were resuspended in EMEM. MNCs were subsequently isolated by gradient centrifugation on Ficoll (Amersham Pharmacia Biotech AB, Uppsala, S) according to the manufacturer's instructions. The MNCs from three animals from the same group were pooled together.

### Determination of memory cells among the isolated MNCs

Isolated MNCs from vaccinated and control mice were explored for the presence of memory cells by determination of antigen-activated T lymphocytes. Half of the MNCs isolated from spleens, bone marrows or peritoneal lavages in concentration 2 × 10^6^/well were stimulated *in vitro *for 5 days with irradiated B16F1 cells (2 × 10^4^/well), while the other half of MNCs (2 × 10^6^/well) were grown for 5 days without additional stimulation. The cytotoxicity on target B16F1 melanoma cells was determined.

### Cytotoxicity assay

For the determination of cytotoxicity of MNCs, the CytoTox^® ^96 non-radioactive cytotoxicity assay (Invitrogen, Carlsbad, California, USA) was used according to the manufacturer's instructions. In brief, 1 × 10^4^/well target cells (B16F1 melanoma cells) were cultured with MNCs at the ratio of 1:10. The release of lactic dehydrogenase (LDH) from the target cells was quantified through measuring of absorption (at 490nm) in formed red formazan crystals. The percentage of cytotoxicity was calculated by the formula: (experimental LDH - LDH in the culture medium)/(total intracellular LDH - LDH in the culture medium) x100 [34]. Experimental LDH release represents LDH values obtained from target B16F1 tumor cells. The results were normalized against control (mock) treated group.

### Statistical analysis

The survival curves of *in vivo *experiments were plotted by the method of Kaplan and Meier using GraphPad Prism 3.0 software. Survival curves were compared using the log-rank test. The p-value < 0.05 was considered as statistically significant. The *in vitro *results were analyzed using SigmaStat 3.0 software. The average mean (AM), standard deviation (SD) and standard error (SE) for each group was determined using descriptive statistics. The differences between the experimental groups were determined using OneWay Anova. The p-value < 0.05 was considered as statistically significant.

## Authors' contributions

PC performed the experiments on animals, carried out cytotoxicity assays, performed the statistical analysis and drafted the manuscript. BJN revised the manuscript and helped to draft the manuscript. VS participated in the experiments on animals and helped to perform the statistical analysis. SN conceived and designed the study, coordinated the study, helped to draft the manuscript and gave the final approval of the version to be published. All authors read and approved the final manuscript.
